# Genome-Wide Identification, Expression Profile of the TIFY Gene Family in *Brassica oleracea* var. *capitata*, and Their Divergent Response to Various Pathogen Infections and Phytohormone Treatments

**DOI:** 10.3390/genes11020127

**Published:** 2020-01-24

**Authors:** Xing Liu, Cunbao Zhao, Limei Yang, Yangyong Zhang, Yong Wang, Zhiyuan Fang, Honghao Lv

**Affiliations:** 1 Germplasm Innovation in Northwest China, Ministry of Agriculture, College of Horticulture, Northwest A&F University, Yangling 712100, Shanxi, China; 13161176649@163.com; 2 Key Laboratory of Biology and Genetic Improvement of Horticultural Crops, Ministry of Agriculture, Institute of Vegetables and Flowers, Chinese Academy of Agricultural Sciences, Beijing 100081, China; cunbaozhao@163.com (C.Z.); yanglimei@caas.cn (L.Y.); zhangyangyong@caas.cn (Y.Z.); wangyong03@caas.cn (Y.W.)

**Keywords:** *Brassica oleracea*, TIFY, JAZ, phytohormone, disease resistance

## Abstract

TIFY, a plant-specific gene family with the conserved motif TIF[F/Y]XG, plays important roles in various plant biological processes. Here, a total of 36 TIFY genes were identified in the *Brassica oleracea* genome and classified into JAZ (22 genes), TIFY (7 genes), ZML (5 genes), and PPD (2 genes) subfamilies based on their conserved motifs, which were distributed unevenly across nine chromosomes with different lengths (339–1077 bp) and exon numbers (1–8). Following phylogenetic analysis with *A. thaliana* and *B. rapa* TIFY proteins, ten clades were obtained. The expression of these TIFY genes was organ-specific, with thirteen JAZ genes and two PPD genes showing the highest expression in roots and leaves, respectively. More importantly, the JAZs showed divergent responses to various pathogen infections and different phytohormone treatments. Compared with the susceptible line, most JAZs were activated after *Plasmodiophora brassicae* infection, while there were both induced and inhibited JAZs after *Fusarium oxysporum* or *Xanthomonas campestris* infection in the resistance line, indicating their probably distinct roles in disease resistance or susceptibility. Further, the JAZs were all upregulated after MeJA treatment, but were mostly downregulated after SA/ET treatment. In summary, these results contribute to our understanding of the TIFY gene family, revealing that JAZs may play crucial and divergent roles in phytohormone crosstalk and plant defense.

## 1. Introduction

The TIFY family is plant-specific and encodes transcription factors (TFs), which was previously annotated as zinc-finger (C-X_2_-C-X_20_-C-X_2_-C) protein expressing in the inflorescence meristem (ZIM) family [1]. *AT4G24470*, which encodes a putative TF that contains the CCT domain and the C2C2-GATA zinc finger domain, was the first gene to be characterized as having a ZIM domain [2]. Then, 29 different *Arabidopsis* loci encoding proteins containing GATA-like zinc fingers were identified by BLAST searches [3]. However, ZIM and two ZIM-like proteins (ZML proteins), ZML1 (AT3G21175) and ZML2 (AT1G51600), belonged to a different group from the other typical GATA-type proteins, as determined by phylogenetic analysis [3,4]. In addition, the ZIM family was uncharacterized in Pfam or InterPro databases, and its definition was confusing [5,6]. In response to these inconsistencies, Vanholme et al. (2007) used ‘TIFY’ instead of ZIM to indicate the most conserved amino acid pattern TIF[F/Y]XG (where X represents any amino acid) [7].

Based on the different domain architectures, the TIFY family was classified into four subfamilies: TIFY, ZML, PEAPOD (PPD), and Jasmonate ZIM (JAZ) [8]. The TIFY subfamily contains only the TIFY domain, while the ZML subfamily also contains the CCT and ZML domains. The PPD subfamily contains a unique N-terminal PPD and a truncated Jas domain, and the JAZ subfamily has the C-terminal Jas domain (also named the CCT_2 domain in Pfam). To date, TIFY genes have been identified in many species, such as 18 TIFY genes in *Arabidopsis* [7], 20 in rice [7], 27 in maize [8], 49 in wheat [9], 18 in pigeon pea [10], 24 in *Populus trichocarpa* [11], and 30 in apple [12]. Moreover, functional research on TIFY family genes has been performed. *ZIM* (*AT4G24470*) has been found to regulate petiole and hypocotyl elongation by mediating cell elongation [4], while *ZML2* (*AT1G51600*) acts as a transcriptional repressor in lignin biosynthesis in maize [13]. *PPD1* (*AT4G14713*) and *PPD2* (*AT4G14720*) are involved in the coordination of leaf growth, and their loss-of-function mutations show leaf enlargement and result in dome-shaped leaves, rather than flat leaves [14]. However, owing to the critical role of JAZ genes in the jasmonic acid (JA) pathway, the JAZ subfamily is clearly the best-characterized member of the TIFY family and has received extensive functional research [15,16].

CORONATINE INSENSITIVE 1 (COI1), the F-box component of the E_3_ ubiquitin ligase complexes (SCF^COI1^), is the primary JA receptor [17,18]. MYC2, the basic helix-loop helix (bHLH) TF, is a key transcriptional activator of responses to JA [19]. However, the link between COI1 and MYC2 was the major question and unidentified until the discovery of the key functions of *AtJAZ1* (*AT1G19180*) [20] and *AtJAZ3* (*AT3G17860*) in JA-mediated responses [21]. JAZ proteins are direct targets of COI1 with the C terminus Jas domain, and JA-Ile or coronatine could promote these interactions and induce the degradation of JAZ proteins by the 26S proteasome in a SCF^COI1^-dependent manner [21,22]. MYC2 also interacted with the C-terminal domain of JAZ proteins and was able to induce a set of JA-responsive genes after the degradation of JAZ proteins [21]. MYC3 and MYC4, two other MYC2-related bHLH TFs, have been proven to be able to interact with the majority of JAZ proteins [23]. In addition to MYC transcription factors, MYB TFs (MYB21/MYB24/MYB75/GL1) targeted by JAZ repressors were identified by yeast two-hybrid screens and confirmed in vivo [24,25].

Brassicaceae (alternative name Cruciferae) is a family with numerous economically valuable members, including vegetables, oil crops, medicinal plants, and ornamental plants. Among these species, genome-wide identification of the TIFY gene family has been carried out in *Arabidopsis* and *Brassica rapa* [7,26]. Cabbage (*Brassica oleracea* var. *capitata* L.), an important Brassica vegetable crop of Cruciferae, is cultivated worldwide. In the field, cabbage inevitably suffers from a variety of diseases. Cluboot caused by the protists *Plasmodiophora brassicae* and Fusarium wilt caused by *Fusarium oxysporum* f. sp. *conglutinans* are major soil-borne diseases in cabbage [27,28], and cabbage black rot caused by *Xanthomonas campestris* pv. *campestris* is a bacterial disease that has caused increasing damage in recent years [29]. In addition, downy mildew, head rot, and soft rot disease also threaten the cultivation of cabbage to some extent [30,31,32]. Recent transcriptome studies have shown that plant hormones participate in the regulation of cabbage disease resistance. Ning et al. (2019) found that the salicylic acid (SA) signalling pathway was induced, while the JA signalling pathway was repressed, in the resistant cabbage genotype after *P. brassicae* infection [27]. Xing et al. (2016) found that the JA and ET signalling pathways and SA-dependent systemic acquired resistance (SAR) play important roles in cabbage resistance to *F. oxysporum* [33].

In this study, the TIFY family genes in cabbage were identified, and their structure, evolution, chromosome location, and expression characteristics were analysed simultaneously. In view of the key roles of JAZ proteins in JA signalling pathway, the expression patterns of cabbage JAZ genes after exogenous phytohormone treatments and inoculation with different pathogens were analysed further. The results of this study contribute to a better understanding of the TIFY gene family, help to reveal the role of JAZ genes in phytohormone crosstalk and plant defense, and provide valuable information for future functional studies.

## 2. Materials and Methods

### 2.1. Identification of the B. oleracea TIFY Family Genes

The genome sequencing of the *B. oleracea* var. *capitata* line 02-12 (02-12 genome hereinafter, http://brassicadb.org/brad/) has been completed [34]. However, according to recent studies, there were many assembly errors and incomplete annotations in the 02-12 genome obtained by next-generation sequencing [35,36,37,38]. Therefore, the newly released genome of the doubled haploid *B. oleracea* var. *capitata* line D134 (D134 genome hereinafter, https://db.cngb.org/search/project/CNP0000469/; manuscript under review) based on the single molecule real-time (SMRT) sequencing methods was used for the identification of *B. oleracea* TIFY family genes.

Based on the annotation information regarding the gene structure, the CDS sequences of all cabbage genes were extracted from the genome and translated into protein sequences by using TBtools [39]. Two methods were used for the identification of TIFY family genes in this work. First, the Hidden Markov Model (HMM) profiles of the TIFY domain (Pfam: PF06200) were downloaded from Pfam (http://pfam.xfam.org/) and used for protein screening in HMMER 3.2.1 (e-value < 0.01) [40]. The first part of the candidate TIFY family proteins was obtained. In addition, BLAST analyses were conducted using 18 *Arabidopsis* [7] and 36 *Brassica rapa* [26] TIFY protein sequences as queries on the D134 genome by using the BLASTP program, thus obtaining the second part of the candidate TIFY proteins in *B. oleracea*. Subsequently, the two candidate sets were merged, the redundant proteins were removed, and their conserved domains were further identified by using NCBI-CDD (https://www.ncbi.nlm.nih.gov/Structure/cdd/wrpsb.cgi) [41]. Finally, cabbage TIFY family genes were obtained from the D134 genome and used for subsequent analysis.

The theoretical isoelectric point (pI) and molecular weight (Mw) of each cabbage TIFY protein were analysed using the ‘Compute pI/Mw tool’ in ExPASy (https://web.expasy.org/compute_pi/). The subcellular locations were predicted using ProtComp 9.0 from Softberry (http://linux1.softberry.com/berry.phtml) and Plant-mPLoc in Cell-PLoc 2.0 (http://www.csbio.sjtu.edu.cn/bioinf/Cell-PLoc-2/).

### 2.2. Chromosomal Location and Tandem Duplication Analysis

Based on the genomic sequence annotation file provided by the D134 genome database, the chromosomal position of the cabbage TIFY family genes were obtained and drew maps using TBtools. MCscanX software (http://chibba.pgml.uga.edu/mcscan2/) was used to search tandem and duplicated genes. The Ka/Ks analysis of the TIFY family genes was also conducted using TBtools.

### 2.3. Gene Structure, Conserved Motif, and Phylogenetic Analyses

To investigate the intron-exon organization of the cabbage TIFY proteins, the coding sequences with corresponding genomic sequences were aligned, and the results were obtained online using the Gene Structure Display Server (GSDS, http://gsds.cbi.pku.edu.cn/index.php) [42]. The conserved motifs in full-length TIFY proteins were identified by MEME (http://meme-suite.org/) [43]. The hylogenetic analysis of *B. oleracea*, *B. rapa*, and *A. thaliana* TIFY proteins was generated by using the Molecular Evolutionary Genetics Analysis software package, Version 6 (MEGA 6) with the neighbour-joining (NJ) algorithm [44]. Bootstrap analysis with 1000 replications was performed to assess group support.

### 2.4. Expression Pattern Analysis of Cabbage TIFY Genes Using RNA-Seq Data

The RNA-seq data of various cabbage tissues (bud, callus, root, stem, leaf, flower, and silique) were downloaded from the DDBJ/EMBL/GenBank Sequence Read Archive (SRA) database (GSE42891) and used for the expression analysis of the cabbage TIFY genes. To determine the expression patterns of JAZ subfamily genes after different pathogen infections, the RNA-seq data sets of cabbage clubroot resistance (XG) and susceptible (JF) lines (28 days after *P. brassicae* inoculation and the control mock-inoculated with sterile water, https://www.ncbi.nlm.nih.gov/sra/SRP144315), and Fusarium wilt resistance (96-100) and susceptible (01-20) lines (0 and 3 days after *F. oxysporum* inoculation, https://www.ncbi.nlm.nih.gov/bioproject/PRJNA548392) were downloaded from SRA database. The data sets of cabbage black rot resistance (Fuji early) and susceptible (87-534) lines (0 and 6 days after *X. campestris* inoculation) were obtained from relevant research in our laboratory (unpublished). All high-quality reads of each sample that passed the quality control were mapped to the *B. oleracea* reference genome, and the uniquely mapped reads were used for expression analysis. The fragments per kb per million (FPKM) method was conducted to normalize and calculate the gene expression levels of TIFY genes in different tissues or JAZ subfamily genes after different pathogen inoculations [45]. The negative binomial (NB) distribution test in the DESeq software package (http://bioconductor.org/packages/release/bioc/html/DESeq.html) was used to test the significance of differences (Fold Changes ≥ 2 and *p*-value ≤ 0.05). The heat maps of hierarchical clustering were constructed in TBtools.

### 2.5. Phytohormone Treatment and Quantitative Real-Time PCR Analysis

The cabbage cultivar ‘Zhonggan No. 21′ provided by the Cabbage and Broccoli Research Group, the Institute of Vegetables and Flowers, the Chinese Academy of Agricultural Sciences (IVFCAAS), was used for the expression analyses after different phytohormone treatments. In a greenhouse, cabbage seedlings were cultivated at 28 °C with 14 h of light/10 h of dark under artificial light until the three-leaf stage. Salicylic acid (SA) and MeJA were dissolved in 100% ethanol, and ethephon was dissolved in sterile distilled water to suitable concentrations as stock solutions and diluted with sterile distilled water containing Tween 20 [0.1% (*v*/*v*)] for foliar spraying. Sterile distilled water containing Tween 20 [0.1% (*v*/*v*)] was used as the mock control. The cabbage seedlings were sprayed with 100 μM SA, 100 μM MeJA and 500 mg/L ethephon. There were three repetitions in every treatment, and each repetition consisted of 10 plants. After two hours, the leaf samples of every treatment were taken and frozen in liquid nitrogen and stored at −80 °C for RNA extraction.

Total RNA of the leaf samples was extracted using a FastPure Plant Total RNA Isolation Kit (Vazyme Biotech Co., Nanjing, Jiangsu Province, China) following the manufacturer’s instructions. The quantity and purity of RNA were estimated using an ND-1000 spectrophotometer (Thermo Fisher Scientific Inc., Wilmington, DE, USA). First-stand cDNAs were synthesized by reverse transcription using HiScript^®^III RT SuperMix (Vazyme Biotech) following the manufacturer’s instructions and diluted to 50 ng/μL for downstream processing. The specific primers of JAZ genes were designed using Premier 6 software. The sequences, amplification length, and locations of each primer have been listed in Table S1, and the specificity of the amplification products was test by agarose gel electrophoresis (Figure S1). Each reaction contained 1.0 μL of cDNA, 0.4 μL of forward and reverse primer (10 μM), 10.0 μL of 2× ChamQ Universal SYBR qPCR Master Mix (Vazyme Biotech), and 8.2 μL double-distilled H_2_O in a total reaction volume of 20 μL and was conducted in a Bio-Rad CFX96 Real-Time PCR System (Bio-Rad Laboratories, Hercules, CA, USA) with three technical replicates by using hard-shell PCR plates (HSP9601, Bio-Rad Laboratories). Conditions for the reaction were as follows: 95 °C for 3 min, followed by 45 cycles of 95 °C for 10 s, 60 °C for 30 s, and 72 °C for 20 s. The delta-delta Ct (2^−∆∆Ct^) algorithm was used to analyse the relative gene expression levels [46,47]. *Actin* (GenBank accession number XM_013731369.1, Table S1) was used as the internal control to normalize the expression of the target genes. Between phytohormone treated and control samples, statistical analysis to find significant differential expression was determined using a two-tailed Student’s *t*-test in Microsoft Office Excel 2017 (*p*-values < 0.05α-level).

### 2.6. Subcellular Localization

The pCAMBIA1300-GFP vector was used for the subcellular localization test and digested with two restriction endonucleases (XbaI and KpnI) to insert the target genes. The CDS sequences of six JAZ genes were amplified with specific primer pairs with homologous arms (Table S2), and the amplification products were recovered using the FastPure Gel DNA Extraction Mini Kit (Vazyme Biotech). Through homologous recombination, the six JAZ genes were connected to the pCAMBIA1300-GFP vector by using the ClonExpress^®^Ultra One Step Cloning Kit (Vazyme Biotech). Then, the recombinant plasmids were transferred into *Agrobacterium tumefaciens* strain GV3101. The transformed *Agrobacterium tumefaciens* was cultured for 24 h at 28 °C in L-broth supplemented with 50 µg/mL kanamycin, sedimented by centrifugation at 5000× *g* for 10 min at room temperature and resuspended in sterile distilled water containing 10 mM MgCl_2_ and 150 µg/mL acetosyringone to an optical density (OD600) of 1.0. After standing for 2 h, cells were infiltrated into the abaxial air spaces of *Nicotiana benthamiana* plants [48]. Forty-eight hours after infiltration, the expression position of the JAZ proteins was observed by a Leica SP8 laser confocal microscope (Leica Microsystems, Inc., Buffalo Grove, IL, USA) using filter blocks to select for spectral emission at 488 nm (matching the GFP), and the empty vector was used as a control.

## 3. Results

### 3.1. Genome-Wide Identification of the TIFY Family Genes in Cabbage

On the basis of HMMER search results, 34 TIFY proteins were identified in the D134 genome (Table S3) and were taken as the first part of the candidate TIFY proteins. Then, 79 and 89 homologous proteins were obtained according to the BLASTP search using 18 *A. thaliana* TIFY proteins and 36 *B. rapa* TIFY proteins (Table S3), respectively. Subsequently, all the candidate TIFY proteins were merged and scanned using NCBI-CDD for the identification of their conserved domains. Finally, a total of 36 non-redundant TIFY genes were identified in the D134 genome of *B. oleracea*, including 22 JAZ, 2 PPD, 5 ZML and 7 TIFY proteins. The gene locus IDs of the 36 TIFY genes in the D134 genome are shown in Table 1, and the homologous loci in the 02-12 genome were present simultaneously (4 TIFY genes have no homologous genes in the 02-12 genome). The nucleotide and amino acid sequences of these TIFY genes are summarized in Table S4. The length of these TIFY proteins ranged from 113 (Boc04g01322) to 359 (Boc03g00474) amino acid (aa) residues with an average length of 236 aa. The molecular weight ranged from 11939.03 Da to 38823.97 Da, and the pI values varied from 4.68 to 10.07. Subcellular location prediction showed that all TIFY proteins were predicted in the nucleus.

### 3.2. Chromosomal Location and Gene Duplication Analysis of the Cabbage TIFY Genes

**Figure 1:**
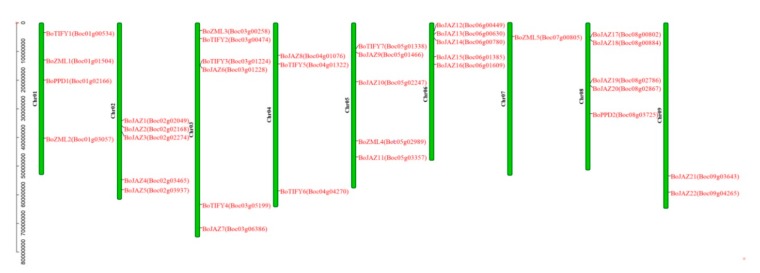
Distribution of TIFY genes on *B. oleracea* chromosomes. Thirty-six TIFY genes (rename and gene locus ID) are shown on the right of each chromosome. Gene positions and chromosome size can be measured using the scale on the left of the figure in mega bases (bp).

All 36 TIFY genes were assigned to nine chromosomes of *B. oleracea* (Figure 1), and the distribution of the TIFY genes on each chromosome was uneven. Chromosome 3 contained the largest number of TIFY genes (6 genes), followed by chromosomes 2, 5, 6, and 8, which contained 5 genes. Only one TIFY gene was located on chromosome 7. Based on the chromosomal location and the subfamily classification, the 36 TIFY genes in *B. oleracea* were renamed (*BoJAZ1*-*BoTIFY7*) (Figure 1 and Table 1).

**Figure 2:**
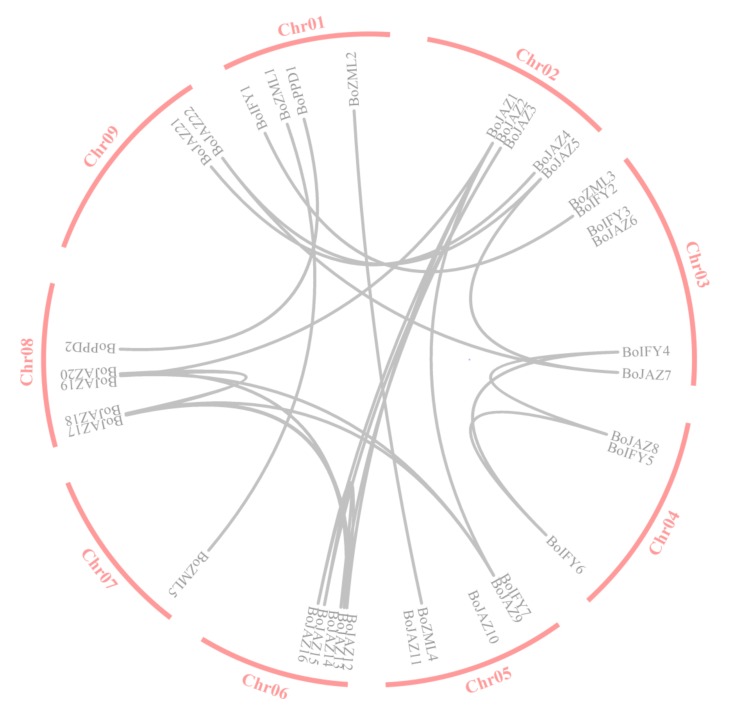
Duplication of the TIFY family genes in *B. oleracea*. The duplicated gene pairs are joined by grey lines.

Gene duplication is one of the most important characteristics of plant genomic structure, which usually contributes to the expansion of gene families. Due to the importance of gene duplication in the evolution of gene families in plants, the duplication patterns of 36 putative TIFY genes were analysed in the cabbage genome. A total of 26 duplicated gene pairs were identified by whole genome duplication (WGD) (Figure 2). The ratios of Ka/Ks can be used as an indicator for the selection pressure of a gene during evolution. The values of all the duplicated TIFY gene pairs were less than 1 in *B. oleracea* (Table 2), indicating that the TIFY genes primarily evolved under the influence of purifying selection.

### 3.3. Gene Structure, Conserved Motif, and Phylogenetic Analysis of the Cabbage TIFY Genes

**Figure 3:**
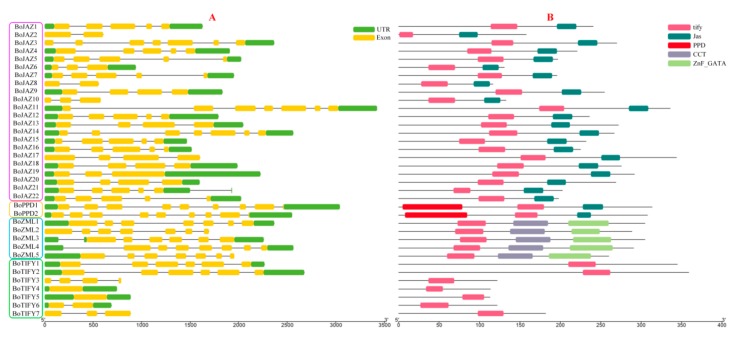
Gene structures (**A**) and motifs (**B**) of 36 TIFY genes identified in *B. oleracea*. UTRs and exons are represented by green and yellow boxes respectively, and introns are represented by grey lines, the length of gene structures can be measured using the scale on the lower in mega bases (bp) (**A**). Boxes with different colors indicate conserved motifs, and the length of motifs in each protein is shown proportionally and can be measured using the scale on the lower in amino acids (aa) (**B**).

The divergence of the exon-intron organization played a critical role in the evolution of multiple gene families. To study the structural diversity of the cabbage TIFY genes, untranslated regions (UTRs), exons and intron organization of each TIFY gene were investigated (Figure 3A). The majority of the TIFY genes contained more than two exons. *BoPPD1* and *BoPPD2* have the largest number of exons at 8, whereas two TIFY subfamily genes (*BoTIFY4* and *BoTIFY5*) had only one exon and no intron. The length and position of exons and introns of cabbage TIFY genes were varied. In addition, the conserved motifs were examined using the MEME motif search tool, five consensus motifs (tify, Jas, PPD, CCT, ZnF_GATA) were detected in the cabbage TIFY genes, and the distribution of these conserved motifs was further analysed (Figure 3B). All 36 cabbage TIFY genes contained the tify motif, and these genes in the same subfamily have consistent motifs. Seven TIFY subfamily genes only contained the tify motif, while five ZML subfamily genes had the extra CCT and ZnF_GATA motifs, 2 PPD genes had the PPD motif, and the largest JAZ subfamily had 22 members with the C-terminal Jas motif.

**Figure 4:**
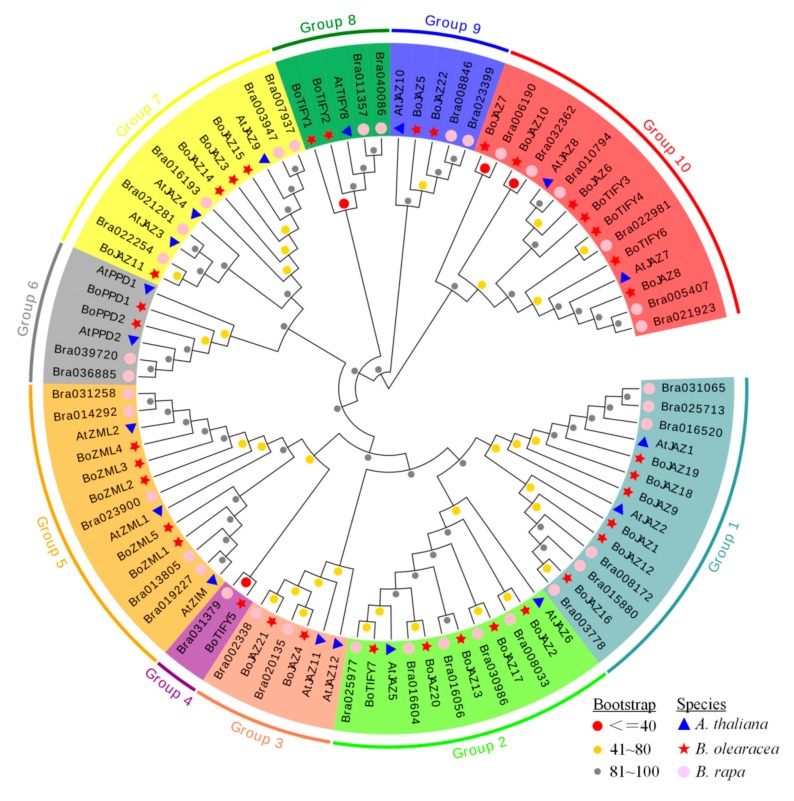
Phylogenetic tree of TIFY genes from *B. olearacea*, *B. rapa*, and *A. thaliana*. The proteins from each species are labeled with different graphics and colors (red star: *B. olearacea*, pink circle: *B. rapa*, blue triangle: *A. thaliana*). The ten groups with different colors represent ten clades. The circles with different colors at the nodes represent bootstrap percentage values (grey: 0–40, yellow: 41–80, red: 81–100) from 1000 replications.

Based on the amino acid sequences of full-length TIFY proteins in *A. thaliana* (18) [7], *B. oleracea* (36) and *B. rapa* (36) [26], the phylogenetic tree was constructed using the neighbour-joining method in MEGA 6 software. The 91 TIFY proteins were grouped into ten clades (groups 1–10 with different background colours) (Figure 4). Among these clades, group 5 was formed with 13 ZML proteins (3 of *A. thaliana*, 5 of *B. rapa*, 5 of *B. oleracea*), and six PPD proteins (2 of *A. thaliana*, 2 of *B. rapa*, 2 of *B. oleracea*) were gathered together in group 6. Groups 1, 3, 7, and 9 were four JAZ subfamily clades, including 14 (2 of *A. thaliana*, 6 of *B. rapa*, 6 of *B. oleracea*), 6 (2 of *A. thaliana*, 2 of *B. rapa*, 2 of *B. oleracea*), 12 (3 of *A. thaliana*, 5 of *B. rapa*, 4 of *B. oleracea*) and 5 (1 of *A. thaliana*, 2 of *B. rapa*, 2 of *B. oleracea*) JAZ proteins, respectively. Groups 4 and 8 were two TIFY subfamily branches, including 2 (1 of *B. rapa*, 1 of *B. oleracea*) and 5 (1 of *A. thaliana*, 2 of *B. rapa*, 2 of *B. oleracea*) TIFY proteins, respectively. However, group 2 and group 10 were two mixed branches, containing both JAZ and TIFY subfamily members. In group 2, there were 11 JAZ subfamily proteins (2 of *A. thaliana*, 5 of *B. rapa*, 4 of *B. oleracea*) and 1 cabbage TIFY subfamily protein (BoTIFY7). In group 10, there were 8 JAZ subfamily proteins (2 of *A. thaliana*, 2 of *B. rapa*, 4 of *B. oleracea*) and 7 TIFY subfamily proteins (4 of *B. rapa*, 3 of *B. oleracea*). The same protein number and similar phylogenetic classification of TIFY proteins in *B. oleracea* and *B. rapa* may reveal the parallel evolutionary relationship of this family between the two species [49].

### 3.4. Expression Patterns of the Cabbage TIFY Family Genes in Various Tissues

**Figure 5:**
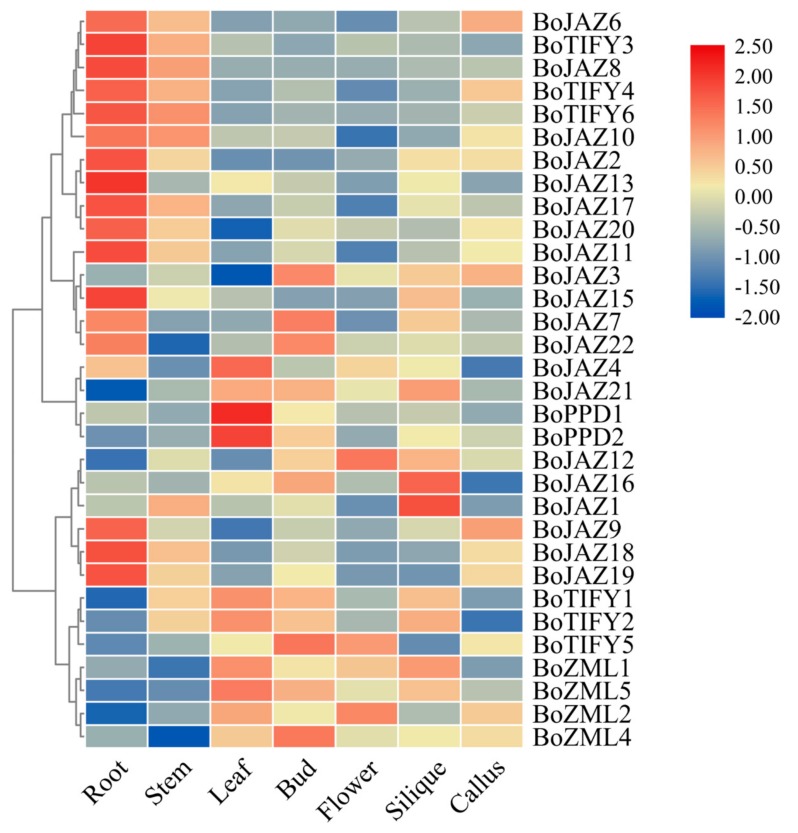
Heat map representation and hierarchical clustering of cabbage TIFY gene expression levels across roots, callus, siliques, stems, leaves, buds, and flowers. Log_2_ transformed values were used to generate the color-coded heatmap, and the color scale with red and blue represent high and low values, respectively, color scale from −2.0 to 2.5.

To explore the expression pattern of the TIFY family genes, RNA-seq data from seven cabbage tissues (root, stem, leaf, bud, flower, callus, and silique) were used (Figure 5 and Table S2). Because these RNA-seq data were obtained from the 02-12 transcriptome sequence data, the four TIFY genes (*BoJAZ5*, *BoJAZ14*, *BoZML3*, *BoTIFY7*) without homologous loci in the 02-12 genome were not analysed. Lower expression levels of ten JAZ genes in three clades (*BoJAZ6*/*BoJAZ8*/*BoJAZ10*, *BoJAZ2*/*BoJAZ13*/*BoJAZ17*/*BoJAZ20*, *BoJAZ9*/*BoJAZ18*/*BoJAZ19*) were observed in leaves, buds, flowers, and siliques, while these JAZ genes showed the highest expression level in roots. *BoJAZ3* and *BoJAZ7* were in the same clade with *BoJAZ15*, *BoJAZ17,* and *BoJAZ22*, while the first two had the highest expression in buds and the last three in roots. In addition, *BoJAZ1*, *BoJAZ12,* and *BoJAZ16* in another clade were highly expressed in silique. For TIFY subfamily genes, the expression levels were diverse in different tissues. Three TIFY subfamily genes (*BoTIFY3*, *BoTIFY4*, *BoTIFY6*) in one clade showed lower expression levels in leaves, buds, flowers, and siliques, while *BoTIFY1*, *BoTIFY2* and *BoTIFY5* in another clade were different. Two PPD genes (*BoPPD1* and *BoPPD2*) showed the highest expression in leaves, indicating that they may be involved in the coordination of leaf growth like *AtPPD1* and *AtPPD2* in *A. thaliana* [14]. In addition, the four cabbage ZML genes may perform similar biological functions with relatively consistent expression levels in all tissues.

### 3.5. Expression Profiles of the Cabbage JAZ Genes induced by Different Pathogen Infection

**Figure 6:**
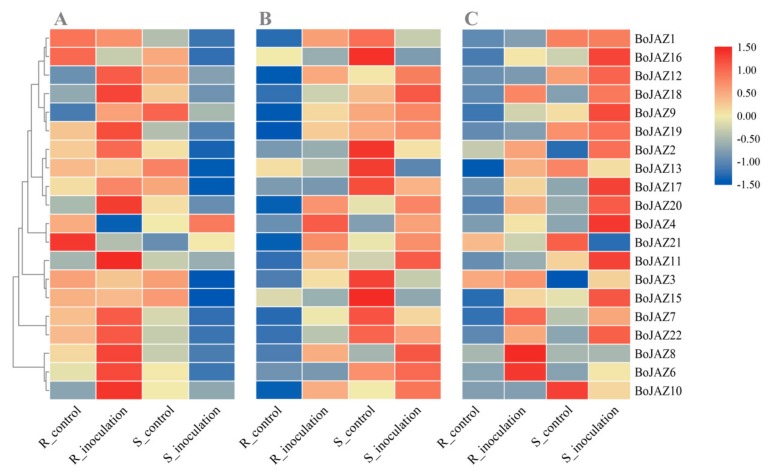
Heat map of cabbage JAZ genes suffering from *P. brassicae*, *F. oxysporum*, and *X. campestris*. (**A**) expression profile of JAZs after *P. brassicae* inoculation; (**B**) expression profile of JAZs *F. oxysporum* inoculation; (**C**) expression profile of JAZs after *X. campestris* inoculation; R: resistant line, S: susceptible line. Log2 transformed values were used to generate the color-coded heatmap, and the color scale with red and blue represent high and low values, respectively, color scale from −1.5 to 1.5.

Jasmonates and related signalling compounds regulate a wide range of biological processes in plants, not only sexual reproduction and development but also host immunity [20,22]. JAZ proteins act as key repressors of JA signalling linking COI1 and downstream transcription factors, suggesting that these proteins may also play key roles in plant defense responses [21,50]. To investigate the expression patterns of cabbage JAZ genes after pathogen infection in both resistant and susceptible materials, RNA-seq data sets of cabbage clubroot, Fusarium wilt, and black rot were used to explore their expression differences. Thirteen JAZ genes were significantly upregulated in the cabbage clubroot-resistant line after *P. brassicae* inoculation, especially *BoJAZ10* (Figure 6A and Table S5). For the susceptible line, in addition to *BoJAZ4* and *BoJAZ21*, other JAZ genes were all downregulated to various degrees, and twelve of them were significantly downregulated, especially *BoJAZ15* (Figure 6A and Table S5). The opposite responses of JAZ genes between cabbage clubroot-resistant line and susceptible line indicate that JAZ genes may play an important role in the resistant reaction of cabbage to *P. brassicae*. Similar to the reaction of the cabbage-resistant line for *P. brassicae*, sixteen JAZ genes in the cabbage Fusarium wilt-resistant line were upregulated after *F. oxysporum* inoculation, and ten of them were significantly upregulated (Figure 6B and Table S5). However, *BoJAZ12* and ten other JAZ genes were also upregulated in the susceptible line. After the inoculation of *X. campestris*, a bacterium causing black rot of cabbage, we also found that many JAZ genes were upregulated in both the resistant line and susceptible line (Figure 6C and Table S5). Although many JAZ genes were induced whether inoculated with *P. brassicae*, *F. oxysporum* or *X. campestris*, the specific JAZ genes were different among them. For example, *BoJAZ15* and *BoJAZ16* were downregulated after *F. oxysporum* inoculation but upregulated after *X. campestris* inoculation. It was suggested that the mechanism of these JAZ members responding to different pathogen infections were different and highly complex, and both redundancy and antagonism were observed.

### 3.6. Expression Patterns of the Cabbage JAZ Genes after Exogenous Phytohormone Treatment

**Figure 7:**
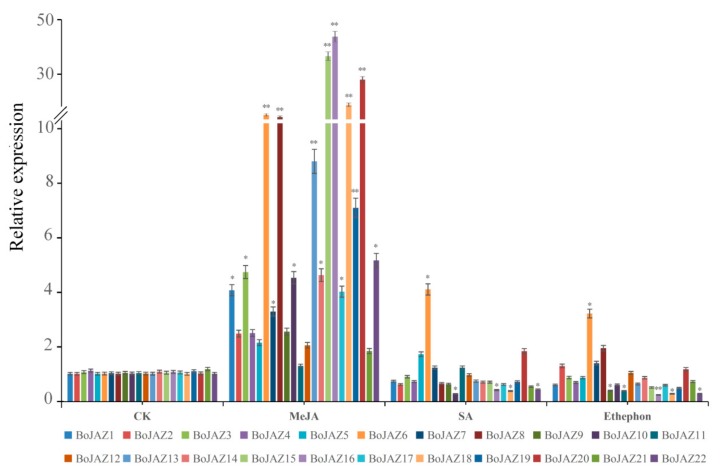
The relative expression of cabbage JAZ genes in the control and exogenous MeJA, SA, and ethylene treatments. Relative expression of JAZ genes was analysed by quantitative real-time qPCR using cabbage actin as a control. Error bars indicate standard deviation, and asterisks indicate significant differences between the control and phytohormone treatment, * *p* < 0.05, ** *p* < 0.01.

Recent studies have revealed that JAZ proteins may play a role in the regulation of diverse phytohormone signalling pathways involved in defence and plant growth [51,52]. In this work, qRT-PCR was conducted to evaluate the responses of cabbage JAZ genes after different phytohormone (JA, SA, ET) treatments. After treatment with MeJA, the transcription of all JAZ genes in cabbage seedlings was induced compared to the control (Figure 7), which was consistent with the results in *A. thaliana* and *B. rapa* [21,26]. Among the JAZ genes, fifteen were significantly upregulated, and six (*BoJAZ6*, *BoJAZ8*, *BoJAZ15*, *BoJAZ16*, *BoJAZ18*, and *BoJAZ20*) were upregulated more than 10-fold, while *BoJAZ11* was upregulated less than twofold and not significantly, showing the different responses of these JAZ genes to MeJA. In response to SA, we found that most cabbage JAZ genes were downregulated, and five JAZ genes (*BoJAZ5*, *BoJAZ6*, *BoJAZ7*, *BoJAZ11*, and *BoJAZ20*) were upregulated. Among these genes, *BoJAZ6* and *BoJAZ10* had the highest and lowest expression levels, respectively. Similar cases also occurred after ethylene treatment. In addition, we found specific *BoJAZ6*, which was concurrently induced by the three phytohormones.

### 3.7. Subcellular Localization of Cabbage JAZ Genes

**Figure 8:**
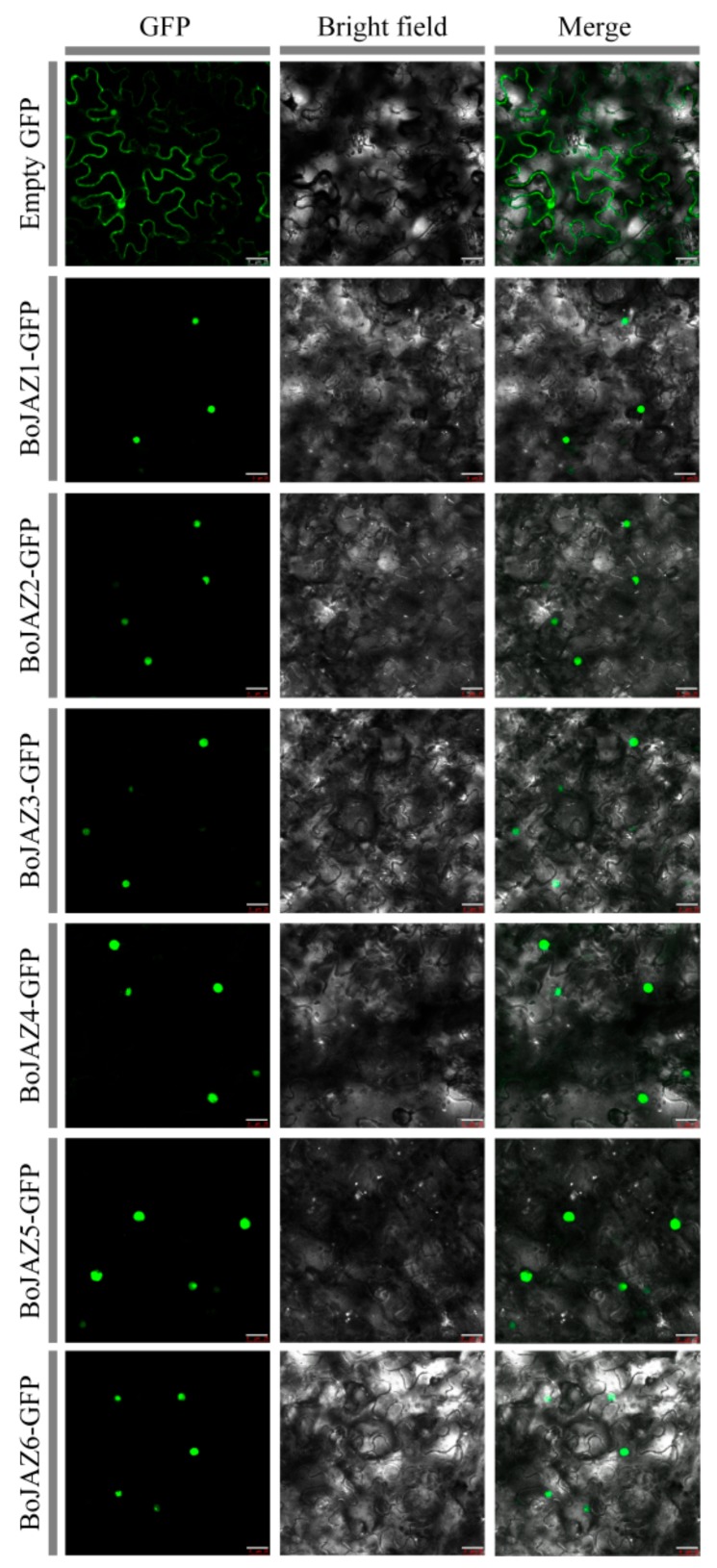
Transient expression of BoJAZ-GFP fusion proteins in tobacco cells. Bars, 25 μm.

Based on the results of the phylogenetic analyses of 91 TIFY genes (Figure 4), twenty-two cabbage JAZ subfamily genes were grouped into six clades (1, 2, 3, 7, 9, 10), and all of them were predicted to be located in the nucleus by using ProtComp and Plant-mPLoc (Table 1). We recombined the pCAMBIA1300-GFP vector with the CDS sequences of six cabbage JAZ genes (*BoJAZ1*, *BoJAZ2*, *BoJAZ3*, *BoJAZ4*, *BoJAZ5*, and *BoJAZ6*), which participated in the six clades of the phylogenetic tree. The GFP signal of all six JAZ-GFP fusion proteins was observed exclusively in the nucleus (Figure 8), which was consistent with the prediction results.

## 4. Discussion

*Arabidopsis thaliana*, one member of the Brassicaceae family, was the first genome sequenced [53]. Subsequently, the whole-genome sequences for other Brassicaceae members were completed, such as *Arabidopsis lyrate*, *Brassica oleracea*, *Brassica rapa*, *Brassica napus*, *Camelina sativa*, and *Raphanus sativus* [54]. The availability of these genomes improved our understanding of phylogenetic relationships in the Brassicaceae family and laid a solid foundation for genome-wide gene identification and functional research [55,56]. Even in closely related species, the genome structure, size and copy number were vary considerably [57], and the variations were restricted to repetitive sequences and affected specific gene families involved in different plant physiological processes [58]. Members of the TIFY family have been demonstrated to be putative TFs with various responsiveness in plant development and defense [59,60]. Hence, we performed genome-wide identification and expression profiling analysis of the TIFY gene family in *Brassica oleracea*. A total of 36 TIFY family genes were identified in the D134 genome of *B. oleracea* and included 22 JAZ, 2 PPD, 5 ZML and 7 TIFY subfamily genes. The total number of TIFY genes and the number of the four subfamily genes in *B. oleracea* were the same as those in *B. rapa*. This result indicated that the evolution rate of the TIFY genes was similar between the two *Brassica* species.

Gene duplication, including tandem, segmental, and whole genome duplication, plays an important role in the evolution of various species and contributes to the expansion of gene families [61,62]. Previous whole genome identification found a total of 1825 gene clusters containing 4365 tandemly duplicated in *B. oleracea*, and there were similar numbers in *B. rapa* (5181) and *A. thaliana* (4170) [34]. In this study, 26 duplicated gene pairs of 36 TIFY genes in *B. oleracea* caused by WGD were identified. WGD is one of the important mechanisms of species evolution, producing new genomic regions and making it more complex and diverse [63]. Similar conditions have been found in other species, such as 19 of the 24 poplar TIFY genes produced by WGD [64]. The large proportion of duplicated genes in the TIFY family revealed that WGD made a large contribution to the generation of the TIFY gene family. Although the duplicated TIFY genes may have a common ancestor, their functions and expression patterns were complex, since duplicated genes can undergo substantial changes in their structures and/or regulatory mechanisms to assume novel roles [62,63]. For example, in the duplicated gene pair of *BoJAZ1*/*BoJAZ19* identified in this work, *BoJAZ1* has five exons with 241 amino acid residues, while *BoJAZ19* has three exons with 292 amino acid residues, and their expression levels were different in various cabbage tissues.

JA regulates large-scale changes in gene expression to exert its many effects in such processes as plant defense response, cell division, photomorphogenesis, and sexual reproduction [65,66]. JAZ proteins repress the activity of transcription factors that execute responses to JA [20,21,22]. JAZ proteins contain two highly conserved sequence regions: the C-terminal Jas domain, which plays a key role in destabilizing the repressor for the response of JA-Ile, and the ZIM/TIFY domain, which mediates homo- and heteromeric interactions between most JAZs [7,67]. However, a non-TIFY JAZ protein (JAZ13, encoded by *At3g22275*) was also demonstrated as a functional repressor of JA-mediated responses in *Arabidopsis* [68]. In this work, 22 JAZ genes were identified in *B. oleracea*, with the largest number being observed among the four TIFY subfamilies, and there were higher expression levels of most JAZ genes in cabbage root compared with other tissues. In view of the important role of JA in regulating root growth [69], we deduced that JAZ proteins may play a key role in this process and other root-related traits.

Phytohormone signalling networks are extensively involved in the process of plant interactions with pests and pathogens, and numerous studies have shown that JAZ targets appear to be mainly TFs associated with hormone regulation, revealing the crosstalk between JA and other plant hormones to some degree [70]. Salicylic acid (SA) is the major signalling molecule associated with the hypersensitive response (HR) and implicated in plant resistance to (hemi)biotrophic pathogens [71], while defenses against necrotrophic pathogens have been linked to JA and ethylene (ET) signalling [72,73]. However, antagonistic relationships between SA and the JA/ET pathway were most often reported [74]. EIN3 and EIL1, two nuclear transcription factors that initiate downstream transcriptional cascades for ET responses, are capable of interacting with JAZ proteins [75]. Meanwhile, EIN3 and EIL1 repressed SID2, a gene encoding an isochorismate synthase required for SA biosynthesis [76]. RGA, a DELLA protein involved in the regulation of gibberellin (GA) signalling, interacted with JAZ proteins to compete for the JAZ-MYC2 interaction, and the ‘relief of suppression’ model was built [76,77,78,79]. In addition, SA-inducible genes were not constitutively expressed in the quadruple-DELLA mutant, indicating that DELLA has a negative effect on SA signalling [80]. In this study, most cabbage JAZ genes were upregulated after MeJA treatment, and different expression levels were also found after SA or ethylene treatment, particularly *BoJAZ6*. It was indicated that the JAZ genes can respond to JA, SA and ET signalling simultaneously. These results further proved that JAZ proteins may play a key role in the crosstalk among different phytohormones, especially JA, SA and ET.

Since phytohormones play a key role in signal transduction when plants encounter pathogens, we investigated the expression of the JAZ genes in cabbage-resistant and cabbage-susceptible lines after pathogen inoculation. We found that many cabbage JAZ genes were induced after inoculation with both fungal and bacterial diseases. The most obvious response was the cabbage response to *P. brassicae*; thirteen JAZ genes were upregulated in the resistant line, and eighteen JAZ genes were downregulated in the susceptible line. The role of SA and JA signalling in the resistance response to biotrophic clubroot has been investigated. In *A. thaliana*, through the expression analysis of SA- and JA-responsive genes, the determination of SA and JA levels and exogenous phytohormone application, both SA and JA pathways were found to contribute to the inhibition of clubroot development [81]. In addition, glucosinolates (GSLs) play roles in plant defense response against microbial pathogens [82]. In Chinese cabbage, the increased content of JA has been proven to mediate the accumulation of aliphatic GSLs and is involved in clubroot during the secondary infection stage [83]. In view of the role of JA in the development of clubroot and the higher expression levels of cabbage JAZ genes in root tissue, and the distinct responses of JAZ genes between cabbage clubroot-resistant and susceptible lines after *P. brassicae* inoculation, functional studies of cabbage JAZ genes for clubroot resistance are warranted. However, the activation of JAZ genes was also observed both in cabbage resistant and susceptible lines after *F. oxysporum* and *X. campestris* inoculation, suggesting that the mechanism by which JAZ genes participate in cabbage disease resistance may be complex and diverse, and there may be functional redundancy and antagonism among them.

## 5. Conclusions

In summary, 36 TIFY genes (22 JAZ, 2 PPD, 5 ZML and 7 TIFY) were identified in the *B. oleracea* genome through genome-wide analysis. The number and length of exons and introns of these TIFY genes were varied, and the conserved motifs of these TIFY genes were consistent in the same subfamily. The expression of these TIFY genes was organ-specific, and a larger number of JAZ genes were activated after different pathogen infections and MeJA treatment. These results presented in this report lay the foundation for further functional characterization of TIFY genes, and improve our understanding of the JAZ genes in plant development and disease resistance through the JA signalling pathway.

## Figures and Tables

**Table 1:** List of the 36 TIFY genes in *B. oleracea.*

	Gene Names	Accession No. ^a^	Homologous Loci ^b^	Chromosome No.	Length (aa)	pI	MW (Da)	Localization Predicted
1	BoJAZ1	Boc02g02049	Bol039351	Chr02	241	9.30	26238.56	N ^c^
2	BoJAZ2	Boc02g02168	Bol034876	Chr02	158	6.16	17527.33	N
3	BoJAZ3	Boc02g02274	Bol041431	Chr02	270	9.96	28913.75	N
4	BoJAZ4	Boc02g03465	Bol036100	Chr02	221	4.97	23089.94	N
5	BoJAZ5	Boc02g03937	-	Chr02	197	9.88	21860.19	N
6	BoJAZ6	Boc03g01228	Bol008534	Chr03	131	9.85	14971.78	N
7	BoJAZ7	Boc03g06386	Bol034224	Chr03	196	9.95	21688.00	N
8	BoJAZ8	Boc04g01076	Bol027372	Chr04	117	9.15	13127.05	N
9	BoJAZ9	Boc05g01466	Bol026828	Chr05	255	9.71	27255.92	N
10	BoJAZ10	Boc05g02247	Bol022524	Chr05	133	9.62	15274.14	N
11	BoJAZ11	Boc05g03357	Bol013829	Chr05	336	9.47	35762.95	N
12	BoJAZ12	Boc06g00449	Bol026137	Chr06	236	9.30	26035.25	N
13	BoJAZ13	Boc06g00630	Bol026339	Chr06	272	9.47	30322.98	N
14	BoJAZ14	Boc06g00780	-	Chr06	267	9.71	28723.60	N
15	BoJAZ15	Boc06g01385	Bol017418	Chr06	232	9.03	25342.54	N
16	BoJAZ16	Boc06g01609	Bol039922	Chr06	225	9.23	24461.58	N
17	BoJAZ17	Boc08g00802	Bol009774	Chr08	344	9.43	38025.74	N
18	BoJAZ18	Boc08g00884	Bol044840	Chr08	276	9.43	30024.88	N
19	BoJAZ19	Boc08g02786	Bol013163	Chr08	292	9.51	31837.28	N
20	BoJAZ20	Boc08g02867	Bol029321	Chr08	269	8.84	29748.45	N
21	BoJAZ21	Boc09g03643	Bol035782	Chr09	203	6.85	21420.92	N
22	BoJAZ22	Boc09g04265	Bol043451	Chr09	198	10.07	21998.47	N
23	BoPPD1	Boc01g02166	Bol014725	Chr01	318	8.64	34439.52	N
24	BoPPD2	Boc08g03725	Bol006854	Chr08	308	8.78	33583.44	N
25	BoZML1	Boc01g01504	Bol009539	Chr01	305	6.05	33079.78	N
26	BoZML2	Boc01g03057	Bol018802	Chr01	289	6.26	31531.93	N
27	BoZML3	Boc03g00258	-	Chr03	305	6.11	33290.86	N
28	BoZML4	Boc05g02989	Bol038395	Chr05	291	6.13	31792.40	N
29	BoZML5	Boc07g00805	Bol042168	Chr07	260	5.74	28003.17	N
30	BoTIFY1	Boc01g00534	Bol017893	Chr01	345	9.51	37246.04	N
31	BoTIFY2	Boc03g00474	Bol017492	Chr03	359	8.49	38823.97	N
32	BoTIFY3	Boc03g01224	Bol016130	Chr03	122	5.06	13390.66	N
33	BoTIFY4	Boc03g05199	Bol036968	Chr03	114	8.97	13033.89	N
34	BoTIFY5	Boc04g01322	Bol014138	Chr04	113	4.68	11939.03	N
35	BoTIFY6	Boc04g04270	Bol037853	Chr04	122	9.10	13653.55	N
36	BoTIFY7	Boc05g01338	-	Chr05	182	8.59	20042.45	N

^a^ The gene locus ID in D134 genome; ^b^ The homologous gene locus in 02-12 genome, ‘-’ represent no homologous gene; ^c^ Nucleus.

**Table 2:** Estimated Ka/Ks ratios of the duplicated TIFY genes in *B. oleracea.*

No.	Paralogous Pairs	Ka ^a^	Ks ^b^	Ka/Ks	Effective Length (bp)	Average S-Sites ^c^	Average N-Sites ^d^
1	BoJAZ1/BoJAZ12	0.136239503	0.466279810	0.292184007	681	157.92	523.08
2	BoJAZ1/BoJAZ9	0.288117541	0.801855456	0.359313564	705	167.17	537.83
3	BoJAZ1/BoJAZ19	0.289411753	0.901233386	0.321128531	702	166.83	535.17
4	BoJAZ3/BoJAZ14	0.090640706	0.237924642	0.380963929	786	184.75	601.25
5	BoJAZ3/BoJAZ15	0.129965792	0.277005584	0.469181128	669	152.92	516.08
6	BoJAZ4/BoJAZ21	0.116023822	0.322633719	0.359614682	600	151.92	448.08
7	BoJAZ5/BoJAZ7	0.116977545	0.248486377	0.470760395	585	141.83	443.17
8	BoJAZ5/BoJAZ22	0.077915489	0.387530995	0.201056147	588	142.08	445.92
9	BoTIFY4/BoTIFY6	0.125567214	0.275793585	0.455294180	318	71.50	246.50
10	BoTIFY4/BoJAZ8	0.128368343	0.326326040	0.393374500	303	69.92	233.08
11	BoJAZ7/BoJAZ22	0.120085286	0.276642241	0.434081524	585	141.92	443.08
12	BoJAZ8/BoTIFY6	0.116309928	0.170137651	0.683622511	348	78.83	269.17
13	BoJAZ9/BoJAZ12	0.291213635	0.704555605	0.413329527	684	157.42	526.58
14	BoJAZ9/BoJAZ18	0.096000993	0.259398924	0.370090173	756	177.08	578.92
15	BoJAZ9/BoJAZ19	0.084821096	0.284963797	0.297655691	741	175.75	565.25
16	BoJAZ12/BoJAZ16	0.185531899	0.474852727	0.390714611	621	138.33	482.67
17	BoJAZ12/BoJAZ18	0.269450506	0.922697085	0.292024881	693	158.08	534.92
18	BoJAZ13/BoJAZ17	0.270636401	1.000601128	0.270473812	795	179.50	615.50
19	BoJAZ13/BoJAZ20	0.254382445	1.085309439	0.234387020	774	173.33	600.67
20	BoJAZ14/BoJAZ15	0.157843966	0.338195612	0.466723875	657	150.00	507.00
21	BoJAZ17/BoJAZ20	0.130211313	0.385659245	0.337633065	804	180.75	623.25
22	BoJAZ18/BoJAZ19	0.082922850	0.304787220	0.272068001	774	181.67	592.33
23	BoTIFY1/BoTIFY2	0.094462148	0.272162609	0.347079816	1014	236.58	777.42
24	BoZML1/BoZML5	0.112145386	0.405464237	0.276585148	774	183.58	590.42
25	BoZML2/BoZML4	0.059319442	0.309010440	0.191965817	849	191.50	657.50
26	BoPPD1/BoPPD2	0.081285177	0.335838405	0.242036575	897	211.17	685.83

^a^ Non-synonymous substitution rate; ^b^ Synonymous substitution rate; ^c^ The average number of synonymous sites; ^d^ The average number of non-synonymous sites.

## Supplementary Materials

The following are available online at https://www.mdpi.com/2073-4425/11/2/127/s1, Table S1: Sequences of 22 specific primer pairs used for qRT-PCR after phytohormone treatment. Table S2: Sequences of six specific primer pairs used for vector construction with homologous arms. Table S3: HMMER and BLASTP search results of the TIFY gene family in B. oleracea. Table S4: Nucleotide and amino acid sequences of 36 TIFY genes in B. oleracea. Table S5: Expression levels of the TIFY genes in different tissues and JAZ genes after pathogen inoculation. Figure S1: Specificity of the qRT-PCR amplifications of JAZ genes.
