# Supplementary figures and images for: Genome-Wide Identification, Expression Profile of the TIFY Gene Family in Brassica oleracea var. capitata, and Their Divergent Response to Various Pathogen Infections and Phytohormone Treatments
Source: Genes (Basel). 2020 Jan 24;11(2):127. doi: 10.3390/genes11020127 (PMC7073855; doi:10.3390/genes11020127)

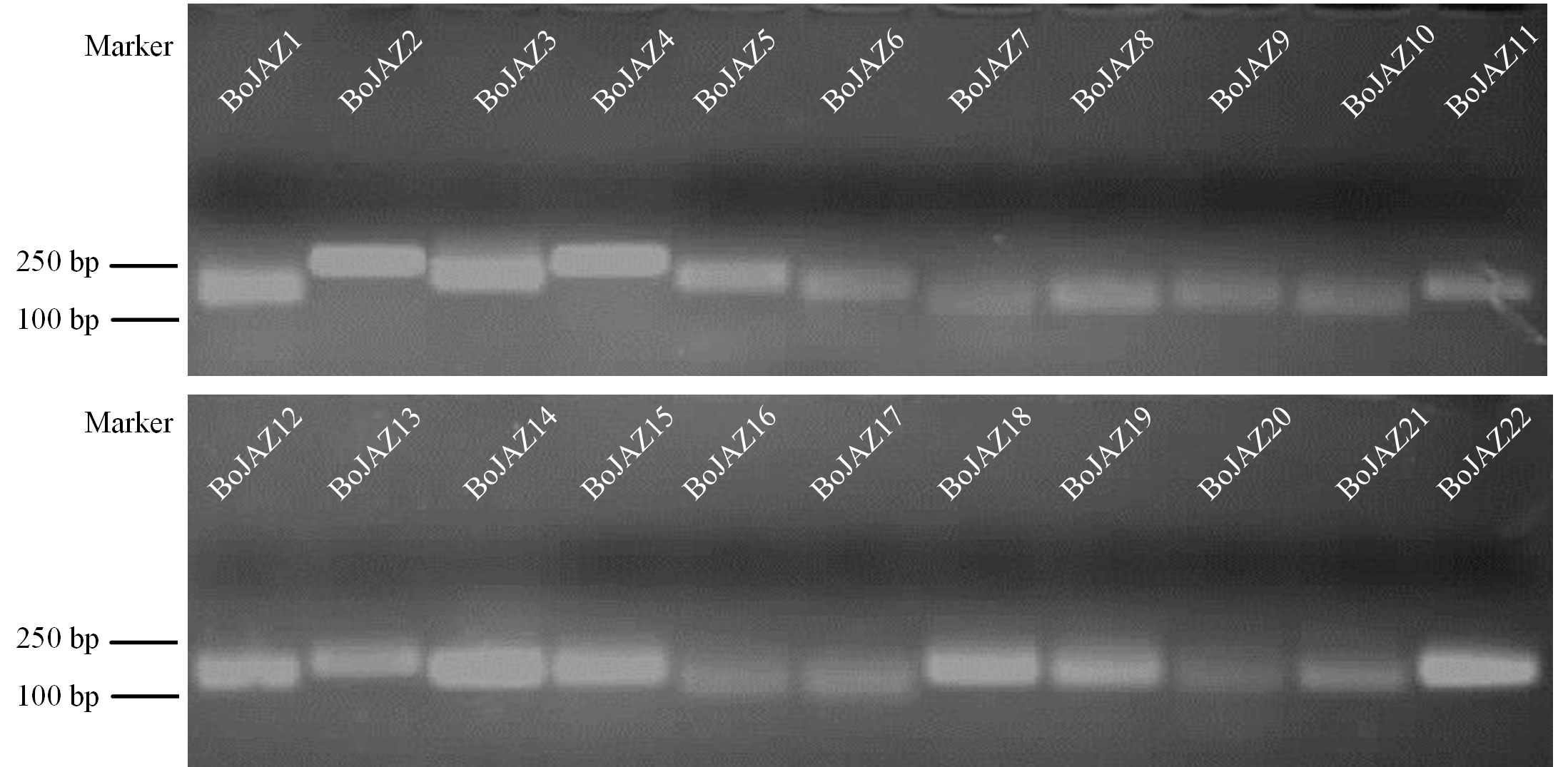

Supplement: Supplementary file 1 [file genes-11-00127-s001.zip › Figure S1.png]
